# Using Acoustic Sensors to Improve the Efficiency of the Forest Value Chain in Canada: A Case Study with Laminated Veneer Lumber

**DOI:** 10.3390/s110605716

**Published:** 2011-05-27

**Authors:** Alexis Achim, Normand Paradis, Peter Carter, Roger E. Hernández

**Affiliations:** 1 Centre de recherche sur le bois, Université Laval, 2425 rue de la Terrasse, QC, G1V 0A6, Canada; E-Mails: normand.paradis.1@ulaval.ca (N.P.); roger.hernandez@sbf.ulaval.ca (R.E.H.); 2 Fibre-Gen, Unit 5 Amuri Park, 404 Barbadoes Street, Christchurch, New Zealand; E-Mail: peter.carter@fibre-gen.com

**Keywords:** acoustic sensors, forestry wood chain, laminated veneer lumber

## Abstract

Engineered wood products for structural use must meet minimum strength and stiffness criteria. This represents a major challenge for the industry as the mechanical properties of the wood resource are inherently variable. We report on a case study that was conducted in a laminated veneer lumber (LVL) mill in order to test the potential of an acoustic sensor to predict structural properties of the wood resource prior to processing. A population of 266 recently harvested aspen logs were segregated into three sub-populations based on measurements of longitudinal acoustic speed in wood using a hand tool equipped with a resonance-based acoustic sensor. Each of the three sub-populations were peeled into veneer sheets and graded for stiffness with an ultrasonic device. The average ultrasonic propagation time (UPT) of each subpopulation was 418, 440 and 453 microseconds for the green, blue, and red populations, respectively. This resulted in contrasting proportions of structural veneer grades, indicating that the efficiency of the forest value chain could be improved using acoustic sensors. A linear regression analysis also showed that the dynamic modulus of elasticity (MOE) of LVL was strongly related to static MOE (R^2^ = 0.83), which suggests that acoustic tools may be used for quality control during the production process.

## 1. Introduction

Acoustic sensors are increasingly being used to improve decision-making in the forest products industry. Recently, they have been applied to a wide array of processes in wood production lines within the mills, such as monitoring the wear of cutting tools [1], estimating surface roughness of the routed wood surface [2,3], or detecting internal decay in glulam (glued-laminated timber) beams [4] and drying cracks on lumber [5].

Early applications of acoustic sensors in the North American forest products industry were also focused mainly on improving the within-mill processes, notably in assessing the performance characteristics of structural lumber [6]. Such applications relied on the fact that in a slender rod of an isotropic and homogeneous material [7], the traveling speed of an acoustic wave can be related to its density and stiffness in the following way:

(1) $$v\;=\;\sqrt{\frac{MOE}{\rho }}$$

where *v* is the speed of sound (m s^−1^), MOE is the (dynamic, *i.e.*, determined in vibratory conditions) modulus of elasticity (N m^−2^), and *ρ* is the density (kg m^−3^). In wood, which is a heterogeneous anisotropic material, acoustic sensors have also been used to determine all elastic constants [8]. Industrially, the longitudinal apparent MOE in static bending is an important wood property as it can influence the value of lumber sold under the machine stress rated (MSR) system [9]. Empirical assessments have shown that a longitudinal stress wave can provide a precise estimation of the apparent MOE in static bending of sawn pieces (which conform to the slender rod assumption) [10,11].

In Canada however, despite an interest for the application of acoustic sensing technology within wood production mills, few applications have been dedicated to improving decision-making higher up in the wood chain, *i.e.*, in the supply chain from the forest to the mill. Such applications could nevertheless bring substantial improvements to the efficiency of the forest industry, because wood is an inherently variable material.

Aside from species-to-species differences [12], the physico-mechanical properties of wood are known to vary within and between individuals of the same species [13]. Within a tree, wood properties normally show a radial progression from pith to bark and along the length of the stem [14,15]. Differences between trees can be attributed to the growing environment (e.g., regional climate or soil characteristics), to the effect of silvicultural practices or to inherent tree-to-tree differences that are often assumed to be of a genetic origin [16]. The latter type is known to represent an important part of the overall variation [17,18]. This implies that even with careful selection of the supply source, there will always remain substantial variability in the properties of the logs that are supplied to a mill. The use of acoustic sensing technology could therefore improve decision-making earlier in the wood chain, either by influencing timber procurement in order to suit the specific needs of a mill, or by helping optimize the use (*i.e.*, finding appropriate applications for end-use and/or improve processing) of timber stored at the mill yard.

Some portable tools have been developed to measure the time-of-flight of an acoustic wave on standing trees [19–21]. Although they were used for several interesting applications, including the detection of decay [22] and insect attacks [23], selection of improved breeding material [24] or stand-level average wood quality assessments [20], these portable tools cannot realistically be used to guide decision making on a tree-by-tree, or log-by-log basis.

Recent developments have lead to the installation of automated prototypes on harvester heads in order to make tree to board predictions [25], but currently most industrial interest is targeted at using resonance acoustic sensors on logs to predict the MOE of sawn lumber [26–28] or other structural wood products. Predictions are based on a measure of the harmonic frequencies of a plane wave induced on the logs, usually by a hammer tap. Using Equation 2, the speed of sound can be derived:

(2) $$v\;=\;\frac{2f_{n}L}{n}$$

where *f_n_* is the natural frequency of the *n*^th^ harmonic of the plane wave signal (Hz) and *L* the log length (m).

For this application of acoustic sensing technology, the MOE of the sawn products is the mechanical property of interest because in several countries lumber used for construction must meet minimum stiffness criteria [29]. However, two problems arise with this estimation. Firstly, as evidenced by Equation 1, an estimation of the dynamic MOE requires a measure of log density, but like other wood properties, wood density varies between and within trees. In addition, the moisture content of the log can vary according to tree characteristics, site and season [30,31], thus affecting the effective log density and, in turn, acoustic velocity [32]. A measure of the dynamic MOE could easily be obtained through adding the use of 3D log scanners (already in place in modern sawmills) [33] and weigh scales to derive an average log density, but to date most industrial applications on logs have relied solely on the acoustic velocity measure.

Secondly, the within-log variation of wood properties results in a variation of MOE among the pieces of lumber produced from a single log. Because the acoustic velocity measured by the resonant frequencies obeys the Law of Mixtures [8] it should approach the volume-weighted average velocities of all lumber pieces sawn from it, a fact confirmed by empirical results [26,27]. However, sawmillers can be more interested in the distribution of the MOEs coming from one particular log, often to identify a threshold acoustic speed for logs yielding a high proportion of structural lumber [28] or to determine how close to the pith to cut structural dimension lumber.

In North America, value can be added through the production of MSR lumber, but the vast majority of the lumber used in construction is still only visually graded [34]. This may explain, on the one hand, why there are still very few reported industrial applications of this acoustic sensing technology in Canada. It is possible that the limited difference in price between MSR and visually graded lumber in recent years may have exacerbated this preference [35].

On the other hand, in recent decades, the forest industry sector in Canada has also seen the development of new engineered wood products, which are increasingly used in residential and commercial constructions [36]. The improved mechanical properties of these products compared to traditional lumber, in addition to their increased uniformity, are the main drivers for their development and adoption in the market [37].

One example is laminated veneer lumber (LVL), which is produced by peeling veneer sheets from logs, drying and gluing them together to a desired thickness before finally sawing lumber pieces from the so-formed sheet. Defects such as knots are no longer forming a continuous zone where mechanical stresses could concentrate. This results in reduced variability and increased mechanical properties in LVL compared with normal dimension lumber. LVL pieces are therefore used as structural elements [36] and their retail price is determined according to their mechanical properties, following a system similar to the MSR grading rules.

Because the wood resource from which they are made is still inherently variable, modern LVL mills are equipped with ultrasonic testing devices that grade veneer sheets [38]. In order to ensure that the overall quality of the product is satisfactory, veneers of different grades are used in combination to produce each piece. However, this post-processing approach leads to important logistical problems in the mill when the log supply produces too few or too many veneers of any particular grade. Typically mills are short supplied on the higher MOE, higher value veneer grades.

This paper describes efforts that were made to use acoustic sensors one step earlier in the LVL forest-to-product value chain. More specifically, this case study was designed primarily to determine if an acoustic sensor applied to logs can help manage the variability of the wood resource in terms of stiffness. As a secondary objective, we also wanted to assess the possibility of using a resonance-based method in order to predict the stiffness of LVL sawn pieces. Provided this could be achieved, the MSR assessment inside the mill could be performed more rapidly and in a non destructive manner.

## 2. Materials and Methods

### 2.1. Acoustic Sensors in the LVL Production Chain

The LVL mill where our study was conducted was equipped with a 2650 DFX Digital Ultrasonic Veneer Tester (Metriguard Inc., USA), which measured the propagation time of an ultrasound wave (UPT, μs) along the length of each veneer sheet. Assuming a constant density, the velocity of the ultrasound can be related to the stiffness of the sheet.

As part of the normal mill operations, each veneer was assigned to one of three possible stiffness grades, *i.e.*, G1, G2 and G3, with G1 being the best (lowest UPT) and G3 the worst. LVL sheets were assembled using a predefined number of veneers from each grade, according to a mill-specific recipe. This commonly applied industrial practice aims to increase the uniformity of the final product and to meet product-specific MOE specifications.

Logistical problems arise when there are too few sheets of one of the grades. The worst problems obviously occur when there is a shortage of the stiffest grade, a factor which can affect the value or even the marketability of the product. Typically, this may happen as a result of processing logs coming from stands where trees show inherently low wood stiffness.

**Figure 1.**
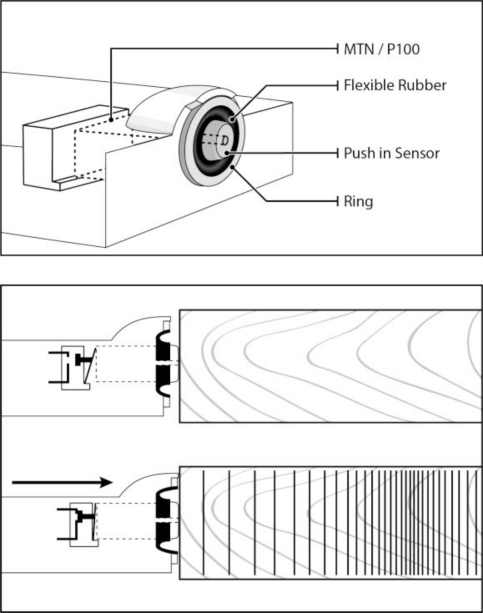
A graphical representation showing how the Monitran P100 accelerometer (MTN/P100) is attached to the push-in sensor of HM200. When the tool is pressed against a piece of wood, the leaf spring located at the back of the accelerometer activates a microswitch to provide electrical power and initiate the processor.

The Hitman HM200 hand tool (Fibre-gen, New Zealand) is used to measure the resonance acoustic velocity on logs. This hand-held tool is equipped with a Monitran P100 accelerometer compliantly mounted in a rubber bossing, which allows it to vibrate freely and thus monitor the oscillation of the cross section of a log which has been tapped with a hammer (Figure 1). The Monitran P100 contains a piezoelectric sensor linked to a charge amplifier. The advantage of using a piezoelectric device is that it provides sturdiness to the HM200 by limiting the movement of components inside the tool. The sensor can make reliable measurements at a wide range of frequencies (1 Hz to 30 kHz) and temperatures (−55 to 250 °C).

Log length is specified by the user and acoustic velocity is derived from harmonic frequencies, which are obtained by performing a Fast Fourier Transformation of the signal [39]. A non contact measurement can also be obtained using a microphone, which records the frequency of sound in the log resulting from the hammer tap. The Hitman LG640 (Fibre-gen, New Zealand) can perform this task automatically on every log entering the mill and enable stiffness-based sorting of logs or peeler blocks for optimised processing.

### 2.2. Case Study

A trial was conducted at a LVL mill located in Amos, Québec (Canada). The mill produced LVL from trembling aspen (*Populus tremuloides* Michx.) which was sourced from various locations in eastern Canada and the North-eastern United States. Two hundred and sixty six logs from 10 different locations were laid out and acoustically tested using the Hitman HM200.

The hypothesis was that acoustic measurements made on logs could be used to split the resource into distinct populations of veneer grade logs. In order to achieve this, acoustic speed was firstly measured on a sample of 50 logs chosen at random within the testing material. Results were then analysed to determine velocity thresholds that would split the population into three classes containing roughly the same number of samples. These were determined to be 3.83 km s^−1^ and 4.04 km s^−1^. Each of the 266 logs were then tested with the Hitman HM200 and classified into a velocity grade. The grades were identified using different colours of spray paint on the cross sections. Red logs were deemed to represent the material with the lowest velocity, with blue logs representing the mid-range and green the highest. Each log grade was processed separately and UPTs on the veneer sheets produced from each subpopulation were recorded. This part of the trial was run on frozen logs, with an outside temperature fluctuating between −15 and −20 degrees Centigrade.

Further, a sample of 35 LVL pieces were destructively tested as part of the normal quality control operations of the mill. These were sawn to average dimensions of 45 × 112 mm with length varying from 2.5 to 3.7 m. The target moisture content was 10%. The MOE in static bending was determined on each LVL piece according to the ASTM D-4761 standard [40]. Prior to this test, acoustic velocity was measured on each sample using the Hitman HM200. The dimensions and mass were also measured so that density could be calculated. An acoustically-derived MOE was calculated using Equation 1 and compared to the static MOE using linear regression.

In order to compare the results with those obtained from solid wood, 15 trembling aspen trees were selected from Laval University’s Montmorency forest, located 75 km north of Québec City. One 2.5 m log was extracted from each tree and all logs were then sawn with a portable sawmill into 73 studs of 50 × 100 mm of cross section (nominally). Each piece was dried to a moisture content of 12% and then tested in static bending following the ASTM D-4761 standard [40]. In order to obtain uniform dimensions and avoid wane, all studs were planed to cross-sectional dimensions of 38 × 89 mm.

## 3. Results and Discussion

### 3.1. Tests on Logs

**Figure 2.**
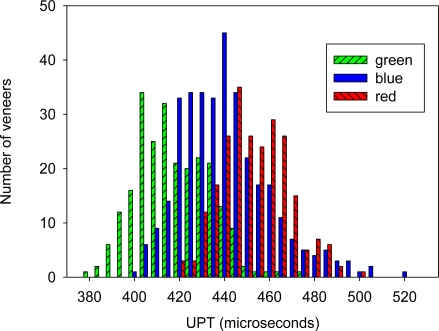
Number of veneers per class of ultrasonic propagation time (UPT). Results are shown for each log grade, with green being from the high velocity logs and red the low.

The three subpopulations of logs defined from our acoustic measurements produced three distinct subpopulations of veneer in terms of quality as assessed by their UPT (Figure 2). The average UPT of each subpopulation was 418, 440 and 453 microseconds for the green, blue, and red populations, respectively. The variation in UPT that remains within each subpopulation can come from several sources, with the most significant being probably the inherent pith-to-bark variability in properties within a log [16]. Despite this observed variation, however, confidence ranges of MOE outturn can be predicted for all three batches of logs.

In mechanically graded lumber, it has been demonstrated that the use of resonant acoustic measurements can yield significant economic benefits despite such uncertainties [27]. Interestingly, the production of LVL implies that the whole cross section of the log is peeled into veneers except for the central core. This should increase the accuracy of the predictions obtained from a resonant acoustic method as, unlike sawn lumber from round logs [26–28,41], almost all the cross sectional area is processed into veneers. In addition, the exclusion of the central core (typically 5 to 8 cm) should reduce the within-log variability because it removes part of the low stiffness wood typically located near the centre of a tree, which is referred to as ‘juvenile’ wood [42]. Conversely, when a log is sawn, cants adjacent to the bark cannot be processed into lumber.

**Figure 3.**
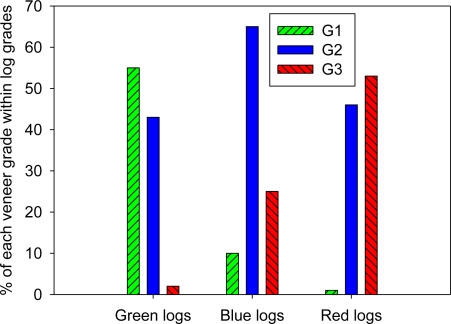
Proportions of veneers of each grade in each log grade. G1, G2 and G3 are veneer grades. Green represents the highest MOE grade and red the lowest.

The range of veneers found in each subpopulation of logs (Figure 3) tends to confirm the potential of the method to improve the efficiency a LVL mill. More specifically, our results show that a resonant acoustic measure can help manage the inventory of veneer sheets of each grade in the mill. For example, assessments using an automated tool taking measurements on the production line, such as the Hitman LG640, could be used to segregate and batch-process logs to manage the within-mill inventory of veneer grades in order to meet specific production targets. These automated measurements can be made online at the mill entrance, segregating out unwanted logs to match the veneer inventory requirements inside the mill. Such a strategy could also be used in conjunction with measurements from hand-held tools in order to monitor the quality of supplies coming from different sources. This would also prove useful for valuing alternative log supplies and guiding wood procurement. Price or maximum transportation distance could be adjusted according to the average wood properties in a particular region or site [20]. Such a system could include the use of standing tree measurements too, although adjustments would be necessary in order to obtain results comparable to those from a resonance tool used on logs [43].

In order to be applicable in Canada, it is necessary to make sure that acoustic assessments remain reliable for a wide range of temperatures, *i.e.*, on frozen or unfrozen logs. Although effective results were obtained in our study, there remain some points of potential concern. Firstly, the Hitman HM200 encountered some difficulties in temperatures well below freezing point. This was possibly related to the hardening of the rubber piece supporting the sensor, to a malfunction of the processor at low temperatures, or simply to a loss of battery power. It would be unlikely to be related to the functioning of the accelerometer itself because it has a working temperature range of −55 to 250 °C. In any case, usage of the hand tool in extremely cold weather could prove problematic. The automated Hitman LG640 would not be affected in the same way, as the sensor in this device is a microphone rather than a rubber-mounted contact sensor, and if required, auxiliary warming of components is a simple matter on an industrial site. Secondly, the velocity threshold between log grades would need to be changed according to temperature. The pattern of acoustic speed variation in logs submitted to a range of temperatures has been described in the literature [32]. The velocity remains rather constant for temperatures well below the freezing point, followed by a rapid decrease in the vicinity of zero degrees (approximately −5 to 5 °C) and a much slower decrease thereafter. Despite the high variation in acoustic speeds, the same study shows that relative differences between logs tested at different temperatures remain almost constant. However, more work needs to be done before the effect of temperature can be fully accounted for.

The prediction of MOE is indeed made more complex by the fact that temperature is not the only variable to consider, as there is also a seasonal variation of moisture content in trees [30,31]. Such variation implies a variation of the effective density in Equation 1, and therefore complicates seasonal adjustments of velocity thresholds. It has been suggested that a simple way around this problem would be to deploy an automated system to measure log density, to which would be added the temperature-specific adjustments. Alternatively, an effective and simpler solution would be to adjust velocity thresholds through a continuous feedback process between the ultrasonic measurements that determine veneer grades and the resonance acoustic measurements made on logs. Such feedback processes are already in place in most mills as part of quality control. For example, in a sawmill, board dimensions are monitored continuously in order to determine the need to adjust band saws.

### 3.2. Tests on LVL

**Figure 4.**
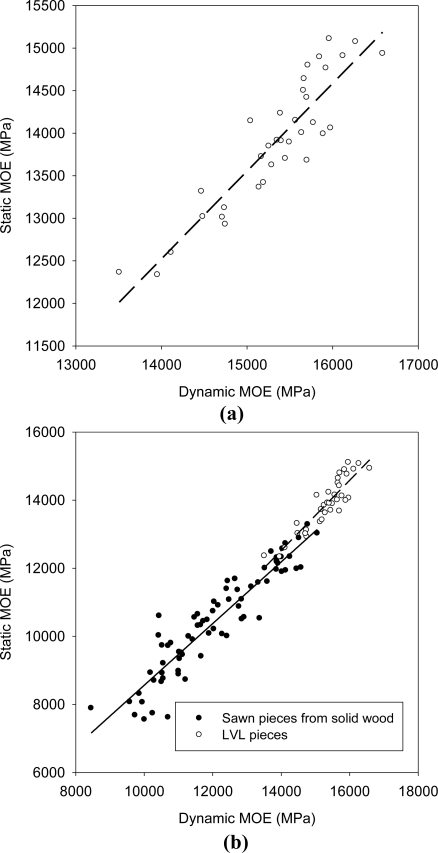
**(a)** Relationship between static MOE measured on LVL pieces and dynamic MOE. R^2^ = 0.83. **(b)** A comparison of the relationships between static and dynamic MOE for traditional sawn lumber (filled circles) and LVL boards (empty circles).

A linear regression analysis showed that acoustic velocity in LVL was weakly related to static MOE (R^2^ = 0.43, data not shown). As observed in Sitka spruce, the strength of the relationship was increased when density was used to calculate the dynamic MOE (R^2^ = 0.83, Figure 4(a)), although its value tended to be higher than the static MOE [44]. The strength of the relationship was somewhat lower than some of the results reported in the literature for sawn pieces of wood [45]. However, when compared with sawn lumber of the same species, the relationship explained a similar percentage of the variation (R^2^ for sawn lumber = 0.86). As the variability in MOE was also much smaller in the LVL sample (Figure 4(b)), we can conclude that the accuracy of the method was high. The resonant acoustic measurement may thus be used in conjunction with a density measurement system in order to predict the stiffness of a LVL board containing veneers of varying qualities. The practical implication is that quality control could be improved during the production process, thereby increasing the speed of testing and reducing the destruction of LVL pieces. It could be argued that the strength of the relationship would be much lower without the presence of low stiffness pieces (MOE < 15,000 MPa, for example), although this is not seen as an issue here since the aim of quality control will be to identify (and possibly discard) such low stiffness pieces, rather than to make a precise assessment of MOE within a given LVL grade.

## 4. Conclusions

Through the example of a LVL production process, this study has shown how a resonance-based acoustic sensor can be used to manage the variability of a wood resource. There are clear benefits that can be drawn from acoustically sorting timber used for the production of mechanically graded products. LVL provides a relevant case in the Canadian context, where traditional dimension lumber is mainly sold using only a visual grading system. The preference for visual grades, and small margin between MSR and visual graded lumber, largely explain why the Canadian forest industry has not yet integrated this technology into its operational strategy. However, with the current economic difficulties of the traditional lumber industries and the resulting interest in value-added products for construction, this situation might be expected to change.

This study also provided the most detailed technical description of the Hitman HM200 hand tool currently available in the scientific literature. Although other types of acoustic sensors may be used in different parts of the forest value chain to increase the traceability of wood properties, harvested stems or cut logs represent the first stage where resonance-based acoustics can be used. Our results and those of similar studies suggest that resonance-based acoustic measurements made on logs at the mill yard would represent a good first step towards improving decision-making early in the wood supply chain. The Hitman HM200 sensor can be used to trace wood properties according to source. In our study, however, we used the Hitman HM200 to simulate what could be achieved using the Hitman LG640, which provides an automated measure of every log entering the wood processing mill.

Industrial applications of this technology in Canada may demand further research with regard to the impact of climatic conditions. Future work with the Hitman HM200 should aim to improve the tool’s performance in cold conditions. The industrial applicability of the resonance-based method for predicting wood properties could be optimized by focusing future research efforts on the seasonal (or in some cases daily) adjustments of acoustic velocity thresholds.
